# Characteristics of the gastrointestinal tract and volatile fatty acids in plateau ruminants

**DOI:** 10.1128/spectrum.01655-25

**Published:** 2026-02-25

**Authors:** Qinran Yu, Chun Huang, Pengjia Bao, Ning Li, Tong Wang, Chaofan Ma, Jingying Deng, Jianlei Jia, Ping Yan

**Affiliations:** 1 Lanzhou Institute of Husbandry and Pharmaceutical Sciences, Chinese Academy of Agricultural Sciences 243814 , Lanzhou, China; 2 College of Agriculture and Animal Husbandry, Qinghai University 207475, Xining, Qinghai, China; 3 Agricultural Genomics Institute at Shenzhen, Chinese Academy of Agricultural Sciences 441191 , Shenzhen, China; 4 Institute of Western Agriculture, Chinese Academy of Agricultural Sciences 838119 , Changji, China; 5 School of Life Sciences, Qilu Normal University 216816, Jinan, China; University of Arkansas Fayetteville, Fayetteville, Arkansas, USA

**Keywords:** gastrointestinal tract, volatile fatty acid, plateau region, ruminant, Pamir yak

## Abstract

High-altitude ruminants face environmental stressors such as low temperature, hypoxia, and short grazing seasons, which challenge energy acquisition and nutrient absorption. The gastrointestinal tract (GIT) plays a central role in metabolic adaptation. In this study, we collected contents from the rumen, reticulum, omasum, abomasum, duodenum, jejunum, cecum, and rectum of Pamir yaks. Microbial composition and metabolic functions across GIT regions were investigated through volatile fatty acid (VFA) determination and 16S rRNA gene sequencing. The results showed that the total VFA concentrations were highest in the forestomach and lowest in the small intestine, with the hindgut showing intermediate levels. Microbial diversity was highest in the reticulum and lowest in the jejunum. At the phylum level, Firmicutes, Bacteroidota, and Proteobacteria were the dominant bacterial taxa. At the genus level, *Prevotella, Rikenellaceae_RC9_gut_group*, *UCG-005*, *Muribaculaceae*, and *Christensenellaceae_R-7_group* were the predominant genera. Functional predictions revealed that the forestomach exhibited a notable enrichment in metabolic pathways associated with carbohydrate, amino acid, and nucleotide metabolism. In contrast, the abomasum and small intestine showed significant enrichment in pathways related to nucleotide and nucleotide-sugar metabolism, protein synthesis, and DNA repair. The hindgut demonstrated enrichment in butyrate metabolism and immune-related pathways. This study highlights the spatial heterogeneity of VFAs, microbial communities, and functional potentials in the GIT, providing theoretical insights into the adaptive mechanisms of energy metabolism in high-altitude ruminants.

**IMPORTANCE:**

This study systematically characterized microbial community composition and volatile fatty acid (VFA) metabolic profiles across different segments of the gastrointestinal tract in plateau ruminants. The results revealed potential metabolic adaptation strategies to extreme conditions such as hypoxia, low temperature, and limited forage availability. The forestomach was identified as the primary site of energy acquisition and showed a marked enrichment of VFA-producing bacteria. Microbial diversity in the small intestine was relatively low, but metabolic pathways associated with nutrient absorption remained highly active. The hindgut exhibited distinct microbial colonization patterns and signs of potential metabolic compensation. Collectively, these findings provided important theoretical insights into nutritional regulation, ecological adaptation, and microecological intervention strategies in plateau ruminants. They also filled a critical gap in high-altitude animal microbiome research and offer significant scientific and practical implications.

## INTRODUCTION

As one of the mammalian groups with the greatest morphological and ecological diversity, ruminants have experienced approximately 40 million years of evolutionary history. Their lineage radiated from primitive herbivorous species, giving rise to a wide range of ecological types, including small-sized carnivorous and omnivorous species (1). To adapt to diverse living environments, different groups of ruminants have gradually developed distinct dietary structures and nutrient utilization strategies. These adaptations have enabled them to thrive across a wide range of ecological habitats, from humid low- to mid-altitude regions to the extremely cold environments of high plateaus (2). Significant interspecies differences in diet composition, morphological traits, physiological regulation, and behavioral patterns have resulted in gastrointestinal microbiota that exhibit high degrees of host specificity and functional diversity (3, 4). Notably, ruminants living in high-altitude environments face the dual challenges of low temperatures and hypoxia. These selective pressures not only reshape the host’s energy metabolism but might also drive adaptive evolution of the gastrointestinal microbiota through host–microbe interactions. This co-evolution likely leads to the formation of microbial communities with distinctive structural and metabolic characteristics (5, 6).

The gastrointestinal tract (GIT) of ruminants exhibits a structurally unique and highly complex ecosystem that exists in close symbiosis with the host, supporting trillions of microorganisms (7–10). These symbiotic microorganisms ferment dietary substrates to produce key nutrients, including volatile fatty acids (VFAs), microbial protein-derived amino acids, and various vitamins. This process plays a critical role in sustaining the host’s energy balance and nutritional metabolism (11, 12). The multichambered stomach and functionally differentiated digestive tract of ruminants provide diverse ecological niches for microorganisms. This structural and functional complexity facilitates the establishment of microbial communities with “regional heterogeneity” in both composition and metabolic characteristics across different anatomical sites (13, 14). These variations correlate closely with local physiological conditions, such as pH, oxygen concentration, substrate availability, and retention time. Such factors directly affect feed conversion efficiency and influence host health and immune homeostasis (15–17). Within this complex system, the rumen, recognized as the largest and most critical fermentation compartment, serves as a “natural bioreactor” for efficient fiber degradation. It contains a diverse array of cellulolytic and hemicellulolytic microbial consortia, significantly enhancing the host’s ability to utilize plant-derived feed (18, 19). Among the fermentation metabolites, VFAs are particularly critical. They serve as the principal source of utilizable energy for the host and contribute to maintaining ruminal pH stability, regulating ruminal motility, and preserving mucosal barrier integrity (20). Relative to the ruminant forestomach, the small intestine was the main site of protein and starch absorption. Both rumen-undegraded protein and dietary protein were efficiently digested and absorbed in this region, with reported digestibility rates of 60%–70% and amino acid absorption rates of 75%–85% (11). As an important secondary fermentation chamber, the cecum further ferments substrates that escape foregut digestion and absorbs residual short-chain fatty acids (SCFAs) and water (21, 22). Therefore, characterizing the GIT microbiota and metabolic profiles of high-altitude ruminants is important. However, current studies have focused on the forestomach, for example, the central role of the rumen in fiber degradation and VFA production (23). Currently, comprehensive studies on the longitudinal spatial succession and functional differentiation of microbial communities along the entire GIT remain limited. And the relationship between the GIT microbiota and metabolic functions in high-altitude ruminants is not yet fully understood.

The Pamir yak (*Bos grunniens*), a characteristic high-altitude ruminant, inhabits the arid desert-steppe ecosystems of the Pamir Plateau at elevations above 3,800 m. As a herbivorous species, it sustains itself mainly on grazed grasses and forbs (24).

However, the nutritional quality and availability of these forage resources vary considerably across seasons, presenting ongoing challenges to the yak’s digestive and metabolic adaptations (25, 26). Benefiting from its distinct genetic background and specialized ecological adaptations, the Pamir yak represents an ideal model organism for studying the co-adaptive relationships between gastrointestinal microbiota and fatty acid metabolism in high-altitude ruminants. Accordingly, this study selected the Pamir yak as the research subject. We employed ultra-high performance liquid chromatography-electrospray ionization-tandem mass spectrometry (UPLC-ESI-MS/MS) combined with 16S rRNA high-throughput sequencing. We systematically analyzed the microbial community structure and VFA metabolic characteristics in multiple key anatomical sites of the Pamir yak, including the rumen, reticulum, omasum, abomasum, duodenum, jejunum, cecum, and rectum. By constructing an integrated “microbiota-metabolite-functional segment” map, we revealed the rules of the GIT microecological succession along the longitudinal axis from the forestomach to the hindgut, as well as the regional differentiation characteristics of metabolic functions. This study not only provides crucial foundational data for an in-depth understanding of the digestive physiological regulation and ecological adaptation mechanisms of high-altitude ruminants in extreme environments but also lays a theoretical basis for research on yak gut microbiota and the formulation of nutritional regulation strategies.

## MATERIALS AND METHODS

### Animals and sample collection

This study was conducted in the natural pasture of Taxkorgan Tajik Autonomous County, Kashgar Prefecture, Xinjiang, China (geographic coordinates: 37.78°N, 75.22°E, approximately 3,800 m above sea level). The experiment was launched on 23 November 2023, and six healthy male yaks grazing in the same pasture were selected as research subjects. These animals had an average initial body weight of 260 ± 13.29 kg and were approximately 3.0 years of age. Immediately after slaughter, the GIT of each yak was dissected using a scalpel, and a total of eight gastrointestinal segments were obtained, including four gastric segments (rumen, reticulum, omasum, and abomasum) and four intestinal segments (duodenum, jejunum, cecum, and rectum). The contents of each gastrointestinal segment were transferred to sterile petri dishes, with the gastric contents being filtered through four layers of sterile gauze to collect the filtrate. Subsequently, the gastrointestinal contents were aliquoted into 5 mL cryovials, flash-frozen in liquid nitrogen, and finally transported to the laboratory for subsequent analysis. In addition, to facilitate data organization and display, the eight GIT segments are abbreviated as follows: rumen (Rum), reticulum (Ret), omasum (Oma), abomasum (Abo), duodenum (Duo), jejunum (Jej), cecum (Cec), and rectum (Rec).

### VFA determination

Liquid chromatography-tandem mass spectrometry (LC-MS/MS) was employed to determine VFA content (27–29). The contents of Rum, Ret, Oma, Abo, Duo, Jej, Cec, and Rec that had been stored at −80°C were thawed on ice. Samples were then subjected to pretreatment and derivatization. Approximately 100 mg of GIT content was transferred to a 2 mL tube containing two small stainless-steel beads. Ice-cold acetonitrile (300 µL) containing the internal standard [^2^H_9_]-pentanoic acid and [^2^H_11_]-hexanoic acid (pre-chilled to 4°C) was added. Samples were bead-milled at 45 Hz for 2 min and sonicated in an ice water bath for 10 min. The mixtures were then centrifuged at 12,000 rpm for 10 min at 4°C. The supernatant was collected and diluted fivefold with 50% acetonitrile containing the same internal standard. Diluted extract of 80 µL was transferred to autosampler vials for derivatization. We sequentially added 40 µL of 200 mM 3-nitrophenylhydrazine (3-NPH) solution (50% acetonitrile/water) and 40 µL of 120 mM EDC-6% pyridine solution (50% acetonitrile/water) to each sample. The mixtures were incubated at 40°C for 30 min, immediately cooled on ice for 1 min, and stored at −80°C until analysis. Quality control samples were prepared by pooling equal volumes of extracts from all samples. All extraction reagents were cooled to −20°C before use. Standards underwent the same pretreatment and derivatization procedures as the samples. Individual stock solutions of each target SCFA were prepared at 1 mg/mL. The stocks were mixed at appropriate proportions and serially diluted to obtain mixed standards at multiple concentrations. For each target VFA, a calibration curve was generated by plotting the ratio of the analyte concentration to internal standard concentration (*x* axis) against the ratio of the analyte peak area to internal standard peak area (*y* axis). Chromatographic conditions were as follows: injection volume 1 µL, flow rate 0.4 mL/min, mobile phase A was 0.1% formic acid in water, and mobile phase B was acetonitrile/methanol (2:1, vol/vol). The gradient elution program was as follows: 0 min A/B (75:25, vol/vol), 2 min A/B (75:25, vol/vol), 11 min A/B (45:55, vol/vol), 12 min A/B (75:25, vol/vol), and 13 min A/B (75:25, vol/vol). Mass spectrometric analysis was performed in the negative electrospray ion mode. Key ion source settings were as follows: ion spray voltage −4,500 V, ion source temperature 450°C, curtain gas pressure 35 psi; nebulizer gas and auxiliary gas both at 50 psi, and collision gas set to the medium mode. Subsequently, the integrated peak areas of the metabolites were substituted into the linear equations of the calibration curves. The resulting values were then used in the quantification equation to obtain the absolute concentrations of each metabolite in the samples. The calculation formula for VFA concentrations in the samples was as follows:


$$Csample\left(ng/g\right)=\frac{N1\times N2\times Cstd\times V}{M}$$

where Csample is the VFA concentration in the sample (ng/g), Cstd is concentration calculated by the standard curve, *V* is the fixed volume (mL), *M* is the sample size (g), *N*1 is the dilution factor, and *N*2 is the conversion factor.

### DNA extraction and PCR amplification

16S rRNA gene sequencing was performed on the 48 GIT content samples. Bacterial genomic DNA was extracted with the DNeasy PowerSoil kit (Qiagen, Hilden, Germany) according to the manufacturer’s instructions, and extracts were stored at −20°C until further use. DNA concentration and purity were assessed with a NanoDrop 2000 spectrophotometer (Thermo Fisher Scientific, Waltham, MA, USA) and by 1% agarose gel electrophoresis. The V3–V4 hypervariable region of the bacterial 16S rRNA gene was amplified using the universal primers 343F (5′-TACGRAGGCAGCAG-3′) and 798R (5′-AGGGTATCTAATCCT-3′) (30). PCRs were set up in a final volume of 25 µL. PCR amplicons were purified with AMPure XP magnetic beads (Beckman Coulter, USA) and quantified using the Qubit dsDNA HS assay. Purified amplicons were pooled at equimolar amounts to construct paired-end sequencing libraries and were sequenced on an Illumina NovaSeq 6000 platform to generate 2 × 250 bp reads.

### 16S rRNA gene sequencing

The raw sequencing data were obtained in FASTQ format. Following sequencing, primer sequences were removed from the raw reads using cutadapt (31). Subsequently, quality control was performed on the trimmed paired-end sequences, including filtering low-quality sequences, denoising, merging pairs, and removing chimeras. These procedures were performed on the QIIME 2 (32) platform using the DADA2 (33) plugin with default parameters. After quality control, amplicon sequence variants (ASVs) and their corresponding abundance tables were generated. Representative sequences for each ASV were extracted in QIIME 2, and taxonomic classification was assigned using the q2-feature-classifier plugin against the SILVA database (version 138). Alpha and beta diversity of the samples were assessed using the QIIME 2 package. Alpha diversity was quantified by the Shannon, Simpson, Ace, and Chao1 indices to evaluate species richness and diversity. Beta diversity was evaluated using principal coordinates analysis (PCoA) based on the Bray-Curtis distance to compare differences in the community composition among samples. The significance of community structure differences was further tested using PERMANOVA (Adonis test). Linear discriminant analysis effect size (LEfSe; LDA > 4, *P* < 0.05) was applied to identify bacterial taxa with significantly different relative abundances between groups, covering taxonomic levels from the phylum to genus. Functional prediction of 16S rRNA gene sequences was performed using PICRUSt2 (version 2.2.0). Differential analysis of predicted functional pathway abundances between groups was conducted with STAMP software. Multiple comparisons were corrected for FDR using the Benjamini-Hochberg method, with FDR-adjusted *P* values < 0.05 considered statistically significant.

### Statistical analysis

Data were presented as mean ± standard deviation (SD), and statistical significance was set at *P* < 0.05. Correlation analyses were performed using Spearman’s rank correlation, and species with correlation coefficients |*r*| > 0.6 and *P* < 0.05 were selected to construct correlation networks. Mantel tests were used to assess the correlation between the Euclidean distance matrix of VFA concentrations across different GIT segments and the Bray-Curtis distance matrix of microbial community structure. Based on the results, correlation networks were constructed to visualize microbial genera significantly correlated with VFAs, categorized as highly significant (*P* < 0.01), moderately significant (0.01 ≤ *P* < 0.05), or nonsignificant (*P* ≥ 0.05) and indicating both the direction and strength of correlations. All figures and statistical analyses were performed using GraphPad Prism (version 5.0) and SPSS (version 20.0).

## RESULTS

### Gastrointestinal VFA analysis

**Fig 1:**
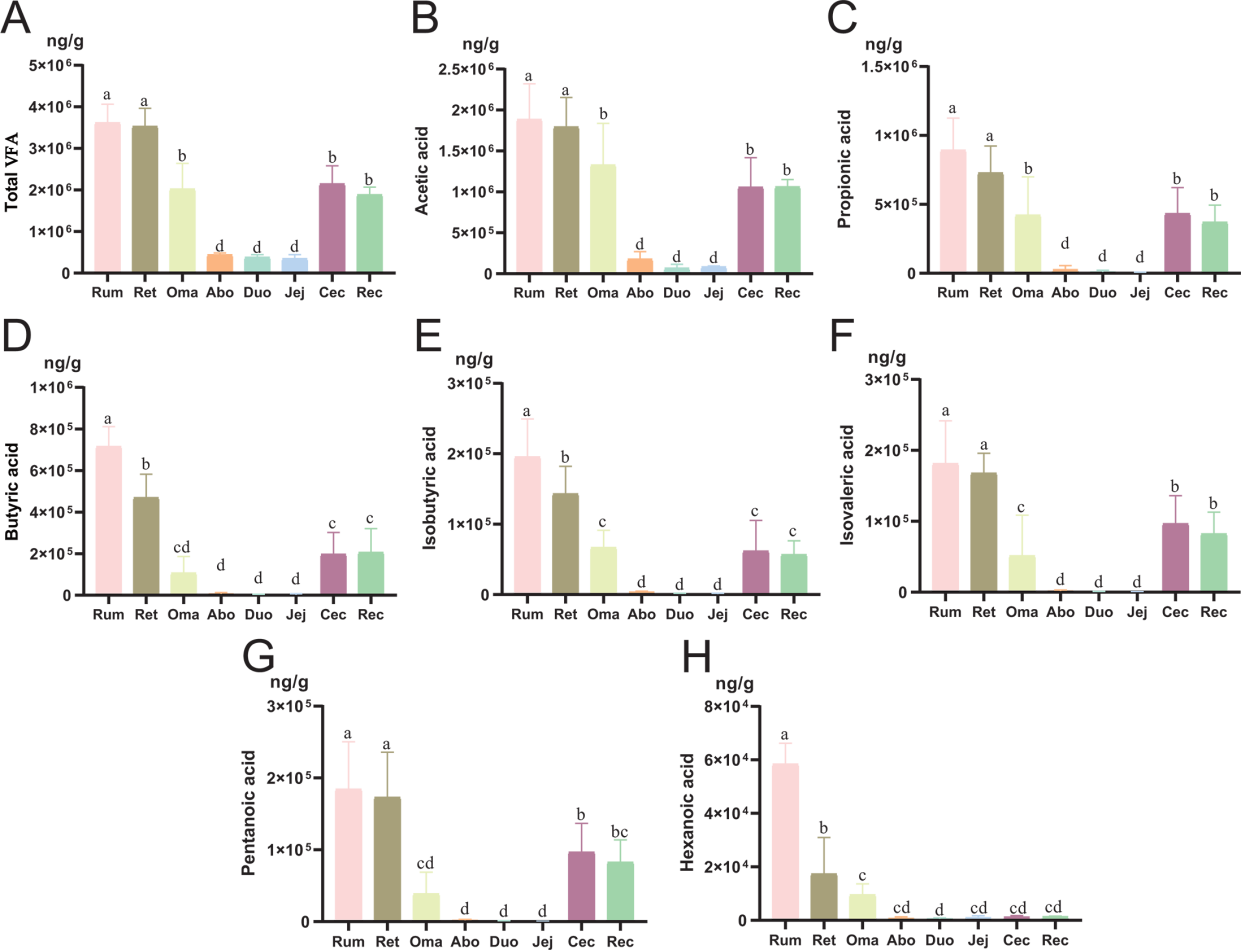
VFA concentrations in different segments of the GIT of Pamir yaks. (**A–H**) Concentrations of total VFAs, acetic acid, propionic acid, butyric acid, isobutyric acid, isovaleric acid, valeric acid, and caproic acid across the GIT. Concentrations are expressed as mean ± SD (*n* = 6 per group). Group differences were analyzed by one-way ANOVA followed by Tukey’s HSD test for multiple comparisons. Different lowercase letters (a, b, c, d) indicate significant differences among groups (*P* < 0.05), while groups sharing the same letter, or including a common letter, are not significantly different. Superscript “ab” indicates that the group is not significantly different from groups labeled “a” or “b” but is significantly different from those labeled “c” or “d.” Similarly, superscript “cd” indicates no significant difference from groups labeled “c” or “d” but a significant difference from those labeled “a” or “b.”

The total VFA concentration, acetic acid, propionic acid, butyric acid, isobutyric acid, isovaleric acid, pentanoic acid, and hexanoic acid in each part of the GIT of Pamir yaks were systematically compared (Fig. 1). The results showed significant differences in VFA concentrations across GIT segments (*P* < 0.05). Total VFA concentration was the highest in the Rum and Ret, followed by the Oma and hindgut, and lowest in the Abo and small intestine (*P* < 0.05). Among them, acetic acid and propionic acid were the main components, and their distribution trends were basically consistent with the total VFA concentration. Butyric acid and isobutyric acid were significantly enriched in the Rum, followed by the Ret (*P* < 0.05). The butyric acid and isobutyric acid in the Rum were significantly higher than those in other parts, followed by the Ret (*P* < 0.05). Isovaleric acid and pentanoic acid were also the highest in the Rum and Ret, followed by the hindgut (*P* < 0.05). In contrast, hexanoic acid was mainly concentrated in the Rum, and its content in the hindgut was significantly reduced (*P* < 0.05).

### Sequencing results and microbial diversity

**Fig 2:**
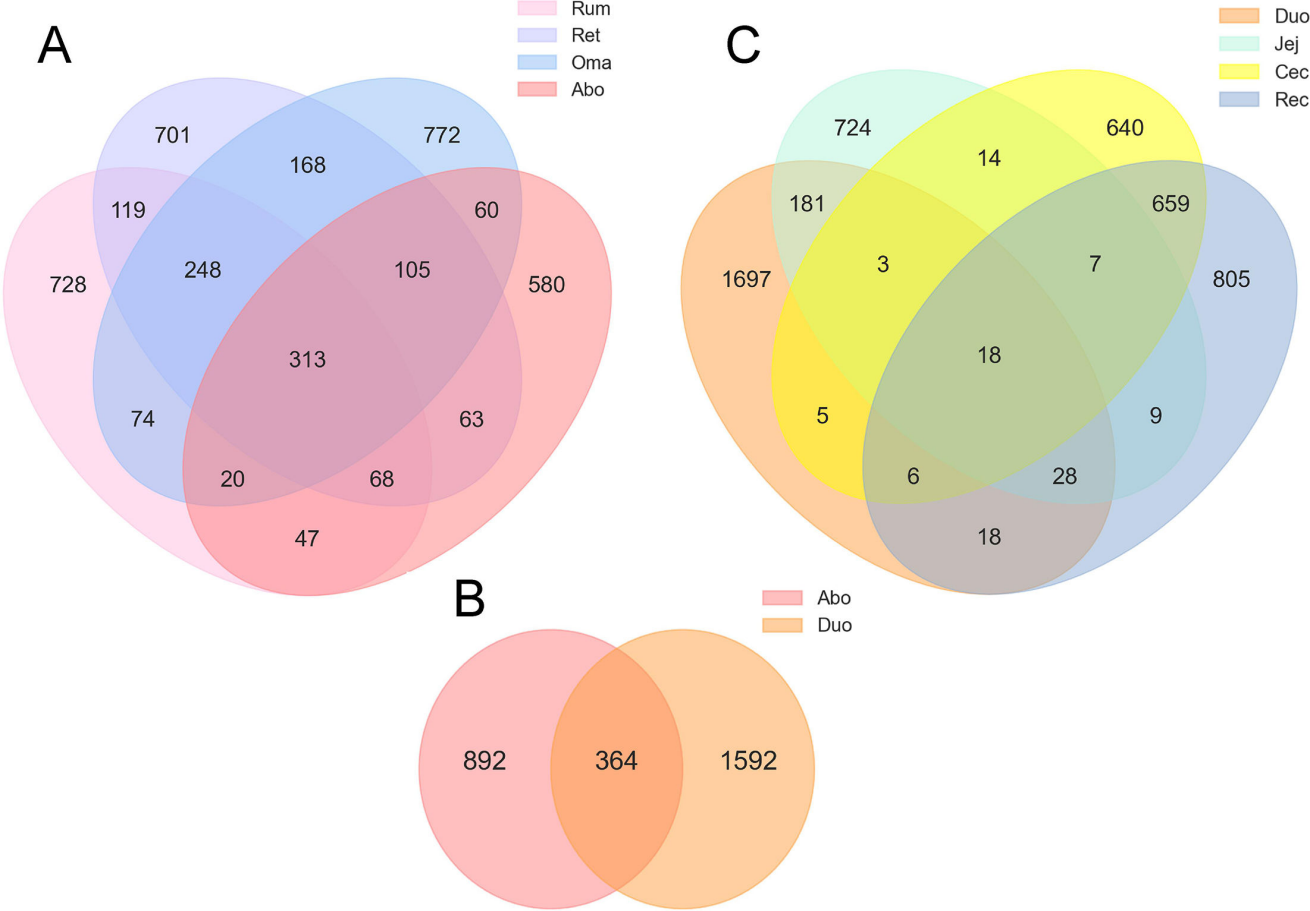
Venn diagrams of ASVs across the GIT segments of Pamir yaks. (**A**) Shared and unique ASVs among the Rum, Ret, Oma, and Abo. (**B**) Shared and unique ASVs between the Abo and Duo. (**C**) Shared and unique ASVs among the Duo, Jej, Cec, and Rec. These diagrams illustrate the overlap and specificity of microbial communities across different digestive tract regions.

After quality control, sequence-directed correction, and primer removal, a total of 2,582,129 valid sequences were obtained, with the number of sequences in each sample ranging from 20,001 to 63,631. The results showed that the Rum and Ret and Ret and Oma shared 748 and 834 ASVs, respectively; the Oma and Abo shared 498 ASVs (Fig. 2A); the Abo and Duo shared 364 ASVs (Fig. 2B); the Duo and Jej shared 230 ASVs; the Jej and Cec shared only 42 ASVs; and the Cec and Rec shared 690 ASVs (Fig. 2C). This suggested that as food moves forward in the digestive tract, the microbial community undergoes distinct spatial differentiation and ecological screening. Further analysis found that the abundance of species in the Duo was the highest, followed by the Ret, Oma, and Rum, and relatively low in the Cec, Abo, Rec, and Jej. The overall results showed that the yak gastrointestinal microbial community had significant regional specificity in spatial distribution, reflecting the differences in the functional positioning of the microbial community at different digestive stages.

To further verify the accuracy of the above analysis, the Shannon, Simpson, Ace, and Chao1 indices were used to evaluate the alpha diversity of each site (Table 1). The results showed that the Shannon index and Simpson index of the Rum were significantly lower than those of the Ret (*P* < 0.05), while the Ace and Chao1 indexes were not significantly different. There were no significant differences in the indexes between the Ret and the Oma (*P* > 0.05). The Shannon, Simpson, Ace, and Chao1 indexes of the Abo were significantly lower than those of the Oma (*P* < 0.01), and the Shannon and Simpson indexes were also significantly lower than those of the Duo (*P* < 0.001). In addition, the alpha-diversity indexes of the Duo and Cec were significantly higher than those of the Jej (*P* < 0.01), while there was no significant difference between the Cec and Rec (*P* > 0.05). The results of this study further confirmed that the yak GIT microbiota showed a trend of diversity gradient distribution along the longitudinal structural axis of the digestive tract.

**TABLE 1:** Analysis of microbial alpha diversity in different sections of GIT of Pamir yak^*a*^

Item	Shannon	Simpson	Ace	Chao1
Rum	8.05 ± 0.48^b^	0.9929 ± 0.0181^b^	580.81 ± 199.24^ab^	582.02 ± 201.71^ab^
Ret	8.68 ± 0.17^a^	0.9961 ± 0.0035^a^	750.21 ± 94.16^a^	757.92 ± 92.95^a^
Oma	8.57 ± 0.22^a^	0.9954 ± 0.0069^ab^	730.85 ± 118.70^a^	742.74 ± 122.43^a^
Abo	7.24 ± 0.17^c^	0.9767 ± 0.0242^c^	524.97 ± 81.20^b^	528.67 ± 81.43^b^
Duo	8.11 ± 0.23^b^	0.9941 ± 0.0071^ab^	491.99 ± 99.32^b^	496.8 ± 100.21^b^
Jej	4.82 ± 0.13^d^	0.9171 ± 0.0263^d^	283.54 ± 48.96^c^	285.32 ± 50.38^c^
Cec	8.26 ± 0.26^ab^	0.994 ± 0.0094^ab^	581.25 ± 108.17^ab^	584.93 ± 107.93^ab^
Rec	8.32 ± 0.54^ab^	0.9937 ± 0.0237^ab^	608.13 ± 186.04^ab^	614.11 ± 190.3^ab^

^*a*^ Note: Shannon and Simpson indices represent microbial diversity, whereas Ace and Chao1 indices represent community richness. All indices were calculated based on 16S rRNA gene sequencing data, and values are expressed as mean ± SD. Different lowercase letters indicate significant differences among groups (*P* < 0.05).

### Classification and composition of gastrointestinal microbial communities

**Fig 3:**
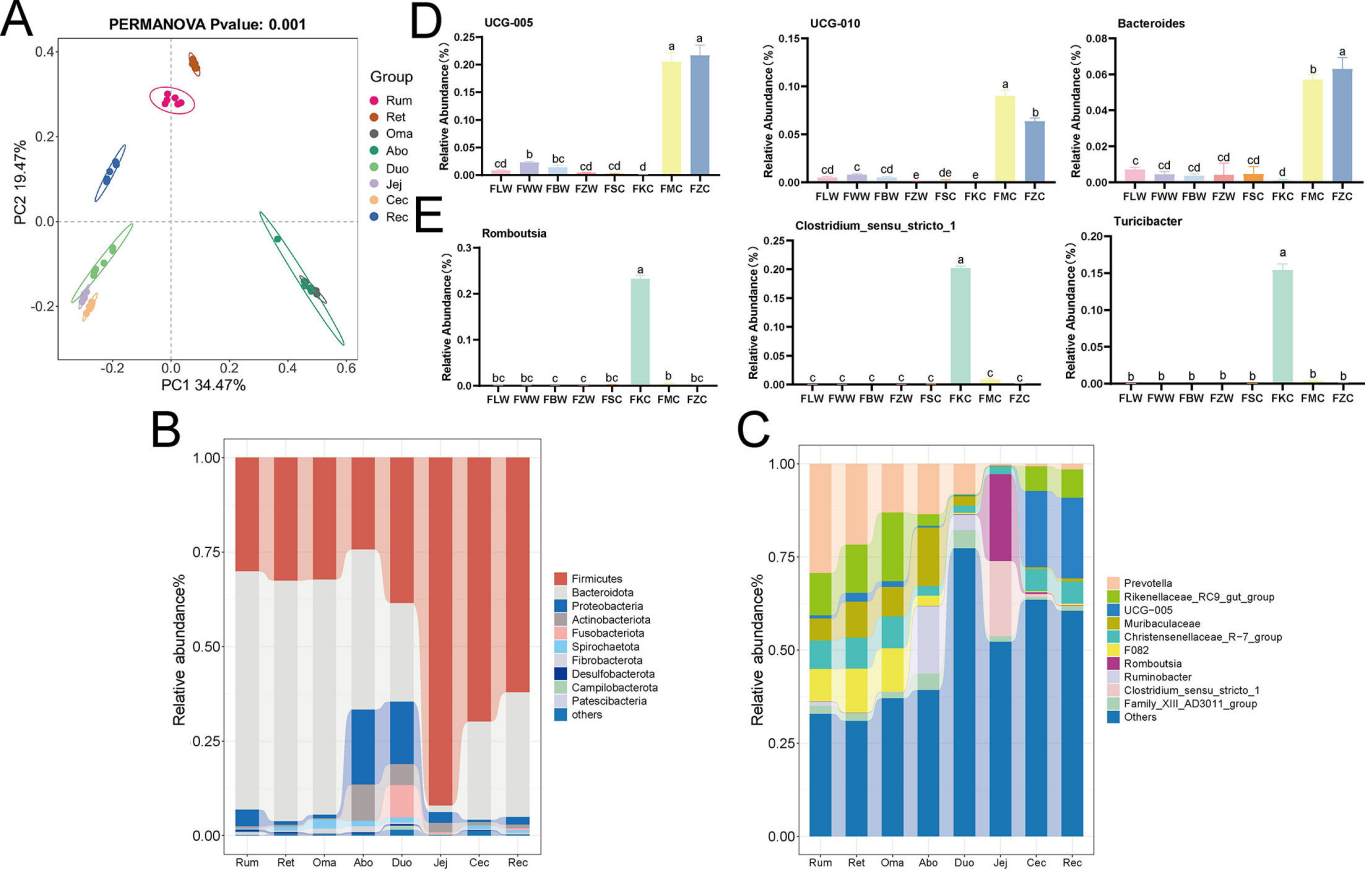
Microbial composition and abundance distribution across the GIT of Pamir yaks. (**A**) Principal coordinates analysis (PCoA) based on Bray-Curtis distances showing differences in the microbial community structure among GIT segments. Statistical significance was assessed by PERMANOVA (Adonis test). (**B**) Relative abundance of microbial communities at the phylum level across GIT segments. (**C**) Relative abundance at the genus level across GIT segments. (**D**) Three dominant genera significantly enriched in the hindgut (Cec and Rec). (**E**) Three dominant genera significantly enriched in the jej. Data are presented as mean ± SD (*n* = 6 per group). Group differences were analyzed by one-way ANOVA followed by Tukey’s HSD test, and significant differences are indicated by different lowercase letters (*P* < 0.05).

Subsequently, we analyzed the microbial community composition of the eight GIT regions. And we found that the Rum-Abo group and the Duo-Rec group were separated, while the microbial composition of the Oma and Abo was similar. Principal component 1 (PC1) and principal component 2 (PC2) explained 22.48% and 11.37% of the variation, respectively (Fig. 3A). The results showed that the microbial community composition in different gastrointestinal regions was different. Subsequently, in the taxonomic composition analysis, we focused on the bacterial taxa at the phylum and genus levels. At the phylum level, all sequences of the 48 samples were identified as 22 phyla, and we found that Firmicutes, Bacteroidota, and Proteobacteria were the dominant bacterial phyla in the eight GITs. We found that Firmicutes were more abundant in the Jej than in other parts of the GIT, followed by the Cec and Rec. Bacteroidota were more abundant in the Rum, Rec, and Oma, while Proteobacteria were most abundant in the Abo and Duo (Fig. 3B). At the genus level, all sequences of 48 samples were identified as 347 genera. As shown in Fig. 3C, the top 10 genera were *Prevotella*, *Rikenellaceae_RC9_gut_group*, *UCG-005*, *Muribaculaceae*, *Christensenellaceae_R-7_group*, *F082*, *Romboutsia*, *Ruminobacter*, *Clostridium_sensu_stricto_1*, and *Family_XIII_AD3011_group*. We found that *Prevotella* was most abundant in the Rum, followed by the Ret, Rum, Oma, and Duo, and its abundance was significantly reduced in the Jej and hindgut (*P* < 0.05). The abundance of *Muribaculaceae* and *F082* in the stomach was significantly higher than that in the intestine, among which *Muribaculaceae* had the highest abundance in the Abo (*P*<0.05), and *F082* had the highest abundance in the Ret and Oma, followed by the Rum and Abo. *Rikenellaceae_RC9_gut_group* was most abundant in the Oma and least abundant in the Duo and Jej. Similarly, *Ruminobacter* was most abundant in the Abo, followed by the Duo. *Christensenellaceae_R-7_group* was most abundant in the Rum, Oma, and Abo, followed by the Cec and Rec, and less abundant in the Abo, Duo, and Jej. *Family_XIIl_AD3011_group* was most abundant in the Abo and Duo. Interestingly, we found that the abundance of *UCG-005*, *UCG-010*, and *Bacteroides* was significantly higher in the Cec and Rec than in other parts of the GIT (*P* < 0.05) (Fig. 3D). The abundance of *Romboutsia*, *Clostridium sensu stricto 1*, and *Turicibacter* was significantly enriched only in the Jej (*P* < 0.05) (Fig. 3E).

**Fig 4:**
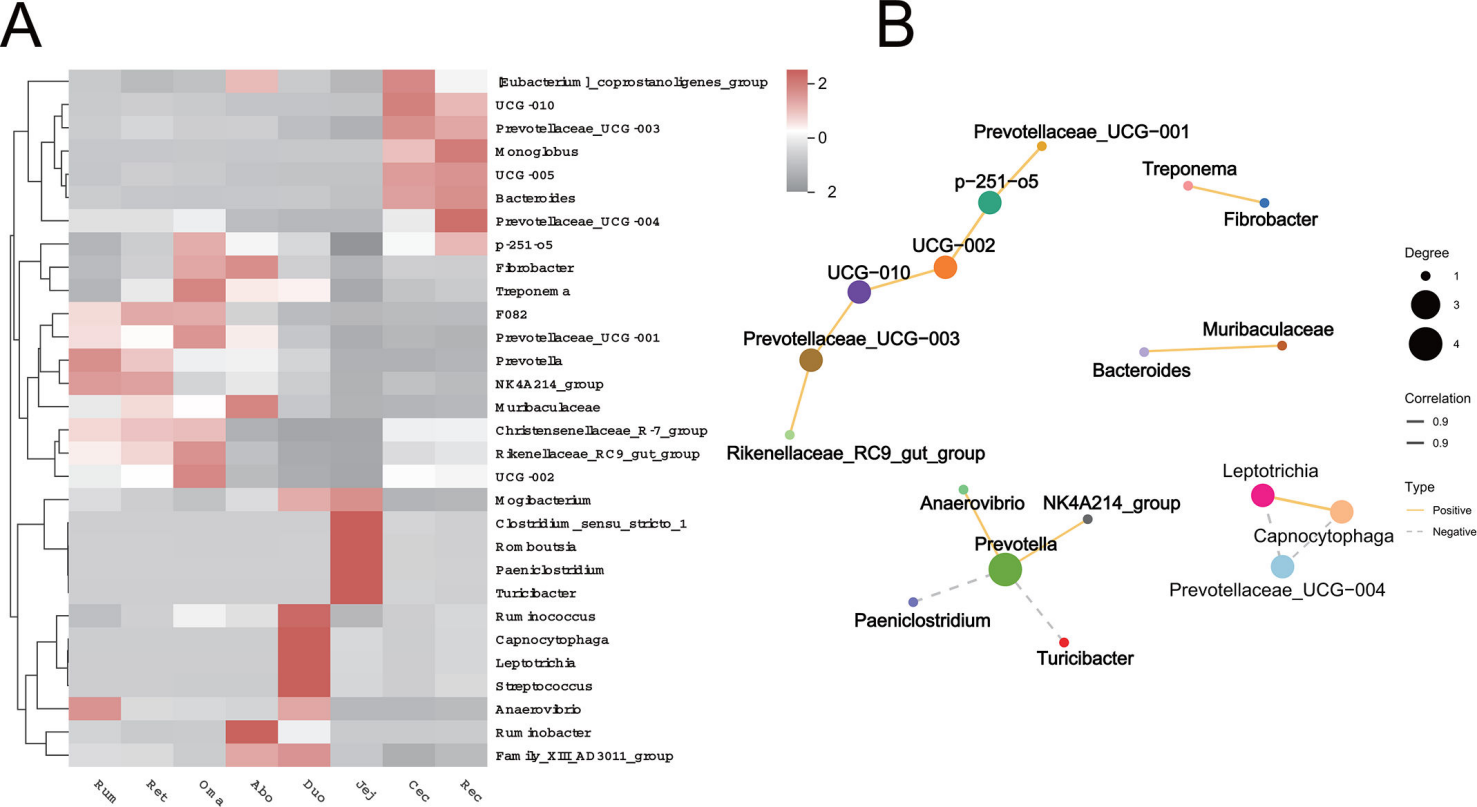
(**A**) Clustering heatmap of the top 30 dominant genera across different GIT segments of Pamir yaks. (**B**) Co-occurrence network of the top 10 dominant bacterial genera in the GIT of Pamir yaks based on Spearman correlation analysis (|*r*| > 0.6, *P* < 0.05). Nodes represent bacterial genera, with node size indicating the degree (number of connections). Edges represent significant correlations between genera, with orange lines indicating positive correlations and gray dashed lines indicating negative correlations.

To further characterize the correlation, diversity, and specificity of microbial communities across the GIT, we selected the 30 most abundant bacterial genera and generated a clustered heatmap to visualize their distribution patterns in each region (Fig. 4A). The results showed that the microbial communities in the different GIT parts showed a clear clustering pattern, reflecting that each digestive tract region has specific microbial composition characteristics. Specifically, *UCG-010*, *Monoglobus,* and *Prevotellaceae_UCG-004* were mainly enriched in the Cec and Rec, exhibiting similar clustering patterns. In contrast, *Clostridium sensu stricto 1*, *Romboutsia*, *Paeniclostridium,* and *Turicibacter* showed high relative abundances exclusively in the Jej. Additionally, *Ruminococcus*, *Capnocytophaga*, *Leptotrichia,* and *Streptococcus* were mainly enriched in the Duo. The forestomach also showed similar clustering.

To further analyze the potential interactions between the dominant bacterial genera in the GIT, we also constructed a co-occurrence network diagram based on the top 30 bacterial genera in relative abundance (Fig. 4B). The results showed that there were three obvious modular structures among bacterial genera, indicating that there may be stable interactions in the microbial community. Among them, *F082*, *Turicibacter*, *Paeniclostridium,* and *Prevotella* exhibited high network centrality. *Treponema* and *Fibrobacter* formed relatively independent subgroups. Additionally, *UCG-010*, *UCG-005,* and *Mogibacterium*, along with their associated genera, also clustered into distinct and relatively independent subgroups.

### Biomarkers in various parts of the GIT

**Fig 5:**
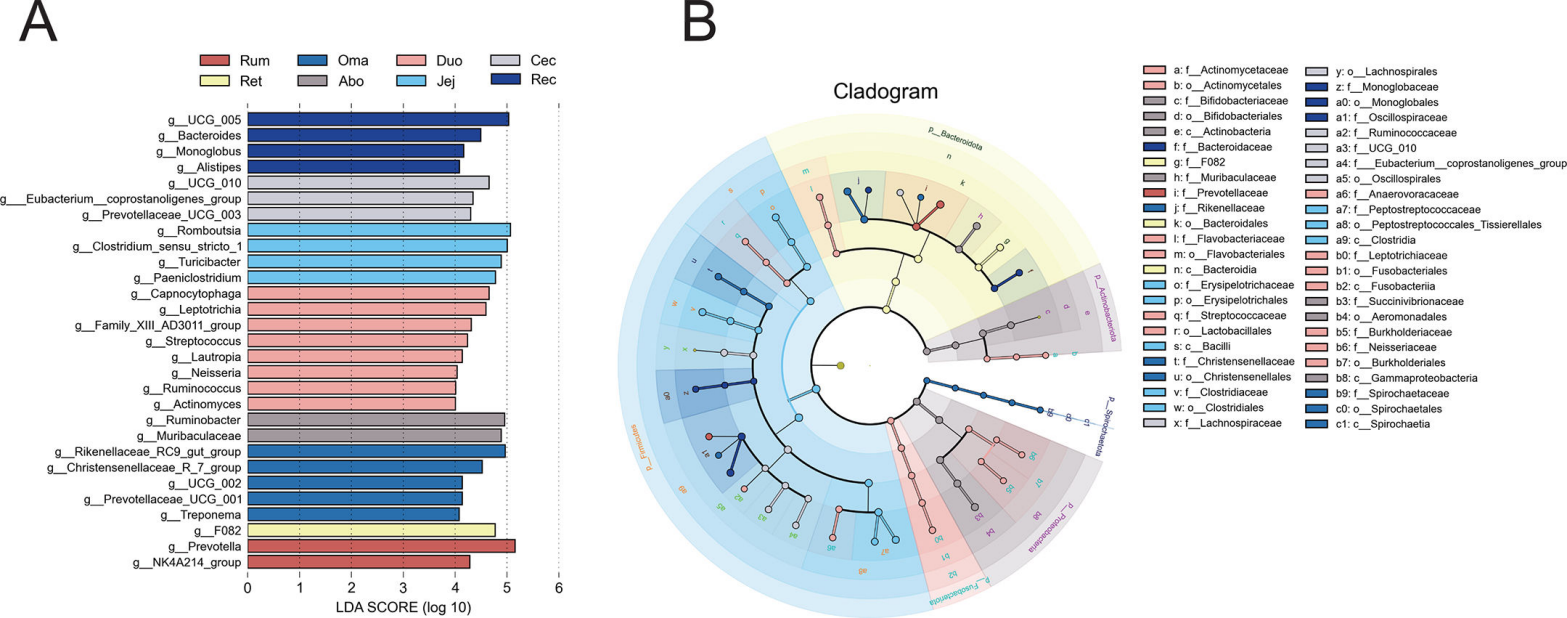
LEfSe results for microbial communities across GIT segments of Pamir yaks. (**A**) LDA bar plot showing taxa with significant differences among GIT segments. The *y*-axis represents genus-level taxa with significant intersegment differences, and the *x*-axis represents their LDA scores (log10); larger LDA values indicate greater discriminatory power. (**B**) Taxonomic cladogram of microbial communities across GIT segments. Node size indicates the average relative abundance of each taxon, and node color indicates the segment in which the taxon is significantly enriched. Hollow nodes represent taxa with no significant differences among segments. Letters annotate the names of microbial taxa that differ significantly between segments.

LEfSe analysis was performed to identify potential microbial biomarkers across different GIT regions (Fig. 5). LDA scores and *P* values (LDA score >4, *P* < 0.05) were used to compare microbial abundances and determine significant differences among groups. The results showed that 2, 1, 5, 2, 8, 4, 3, and 4 genus-level biomarkers were identified in the Rum, Ret, Oma, Abo, Duo, Jej, Cec, and Rec, respectively. The Rum group was mainly characterized by *NK4A214_group* and *Prevotella* biomarkers, *F082* was significantly enriched in the Ret, and the Oma was dominated by *Christensenellaceae_R_7_group*, *Rikenellaceae_RC9_gut_group*, *Treponema*, *Prevotellaceae_UCG_001,* and *UCG_002* microorganisms. *Ruminobacter* and *Muribaculaceae* were significantly enriched in the Abo. Among them, the most enriched bacteria were *Capnocytophaga*, *Ruminococcus*, *Leptotrichia*, *Actinomyces*, *Lautropia*, *Family_XIII_AD3011_group*, *Streptococcus*, and *Neisseria* in the Duo.

The Jej was dominated by four microorganisms: *Romboutsia*, *Turicibacter*, *Clostridium_sensu_stricto_1*, and *Paeniclostridium*. Subsequently, we found that *UCG_010*, *Prevotellaceae_UCG_003*, and *Eubacterium_coprostanoligenes_group* were significantly enriched in the Cec. *Monoglobus*, *Bacteroides*, *UCG_005*, and *Alistipes* were significantly enriched in the Rec.

### Functional gene prediction analysis based on PICRUSt

**Fig 6:**
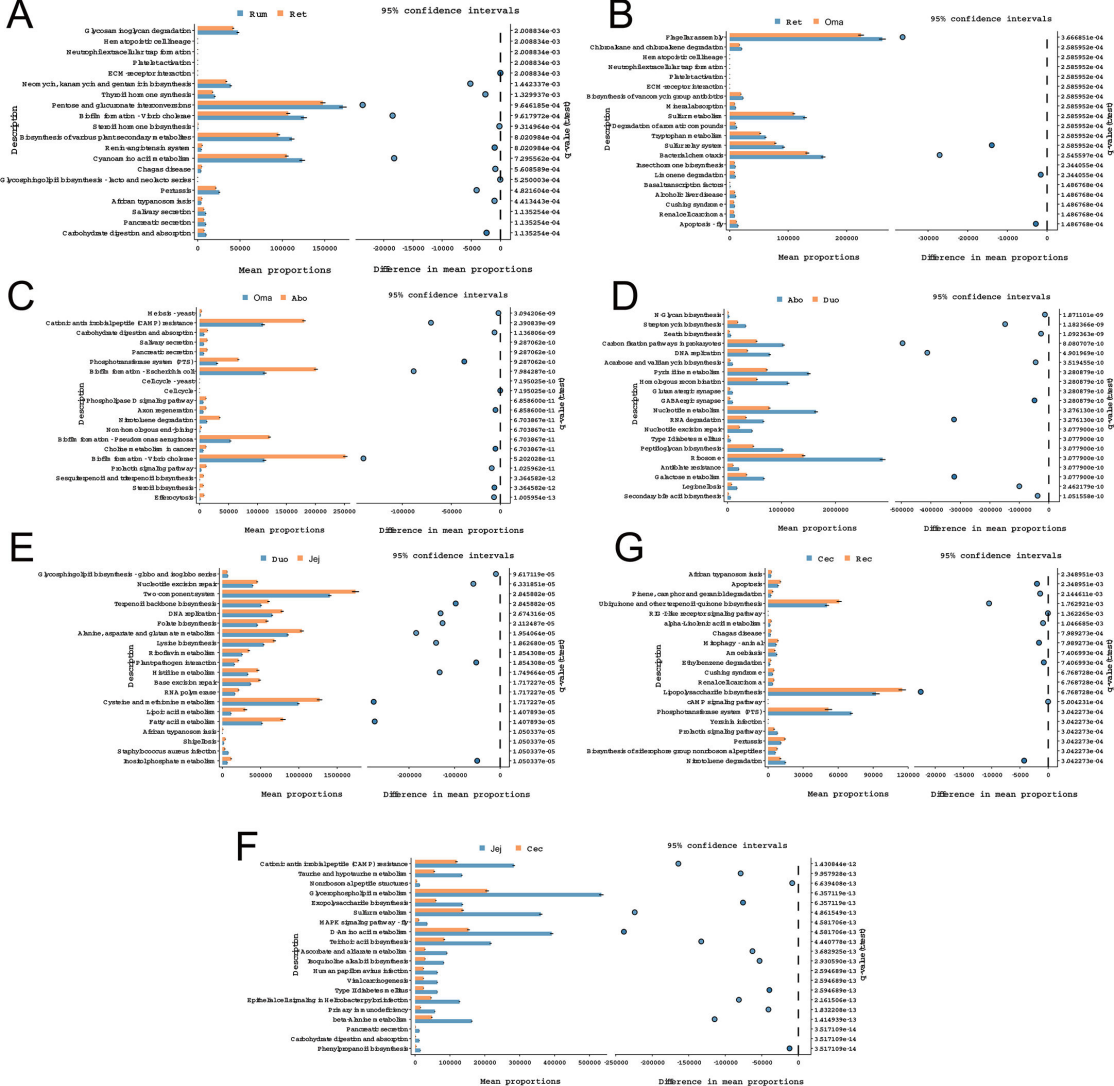
Enrichment analysis of KEGG metabolic pathways between adjacent GIT segments. (**A**) Rum vs Ret; (**B**) Ret vs Oma; (**C**) Oma vs Abo; (**D**) Abo vs Duo; (**E**) Duo vs Jej; (**F**) Jej vs Cec; (**G**) Cec vs Rec. Functional gene abundances were predicted using PICRUSt, and the significance of differential pathways was assessed by *t*-test with FDR correction (*q* < 0.05). Dot color represents fold change, and dot size represents statistical significance (−log10 FDR value).

Based on functional predictions from PICRUSt2, microbial functional pathways were compared across the different GIT regions of yaks (Fig. 6). The analysis was performed at KEGG Level 3 to obtain a more detailed functional resolution, and the top 20 significantly enriched pathways were identified (*P* < 0.05, FDR-corrected). The results revealed 21 significantly enriched pathways during the transition from the Rum to the Ret (*P* < 0.001) (Fig. 6A). Among them, the relative abundances of pentose and glucuronate interconversions, biosynthesis of various plant secondary metabolites, cyanoamino acid metabolism, and glycosaminoglycan degradation were significantly increased in the Ret (*P* < 0.001). During the transition from the Ret to the Oma, the flagellar assembly, bacterial chemotaxis, and sulfur metabolism pathways were significantly enhanced (*P* < 0.001) (Fig. 6B). From the Oma to the Abo, significant reductions were observed in pathways related to biofilm formation, *Vibrio cholerae*; biofilm formation, *Pseudomonas aeruginosa*; biofilm formation, *Escherichia coli*, and cationic antimicrobial peptide (CAMP) resistance (*P* < 0.001) (Fig. 6C). During the transition from the Abo to the Duo, pathways such as ribosome, nucleotide metabolism, and pyrimidine metabolism were markedly enriched (*P* < 0.001) (Fig. 6D). From the Duo to the Jej, the two-component system, alanine, aspartate and glutamate metabolism, and cysteine and methionine metabolism pathways were significantly reduced (*P* < 0.001) (Fig. 6E). In the transition from the Jej to the Cec, we observed a striking increase in glycerophospholipid metabolism, sulfur metabolism, D-amino acid metabolism, and beta-alanine metabolism (*P* < 0.001) (Fig. 6F). Finally, during the Cec-to-Rec transition, the phosphotransferase system (PTS) pathway was significantly decreased, whereas lipopolysaccharide biosynthesis and ubiquinone and other terpenoid-quinone biosynthesis were significantly increased (*P* < 0.001) (Fig. 6G).

### Correlation analysis between VFAs and microorganisms

**Fig 7:**
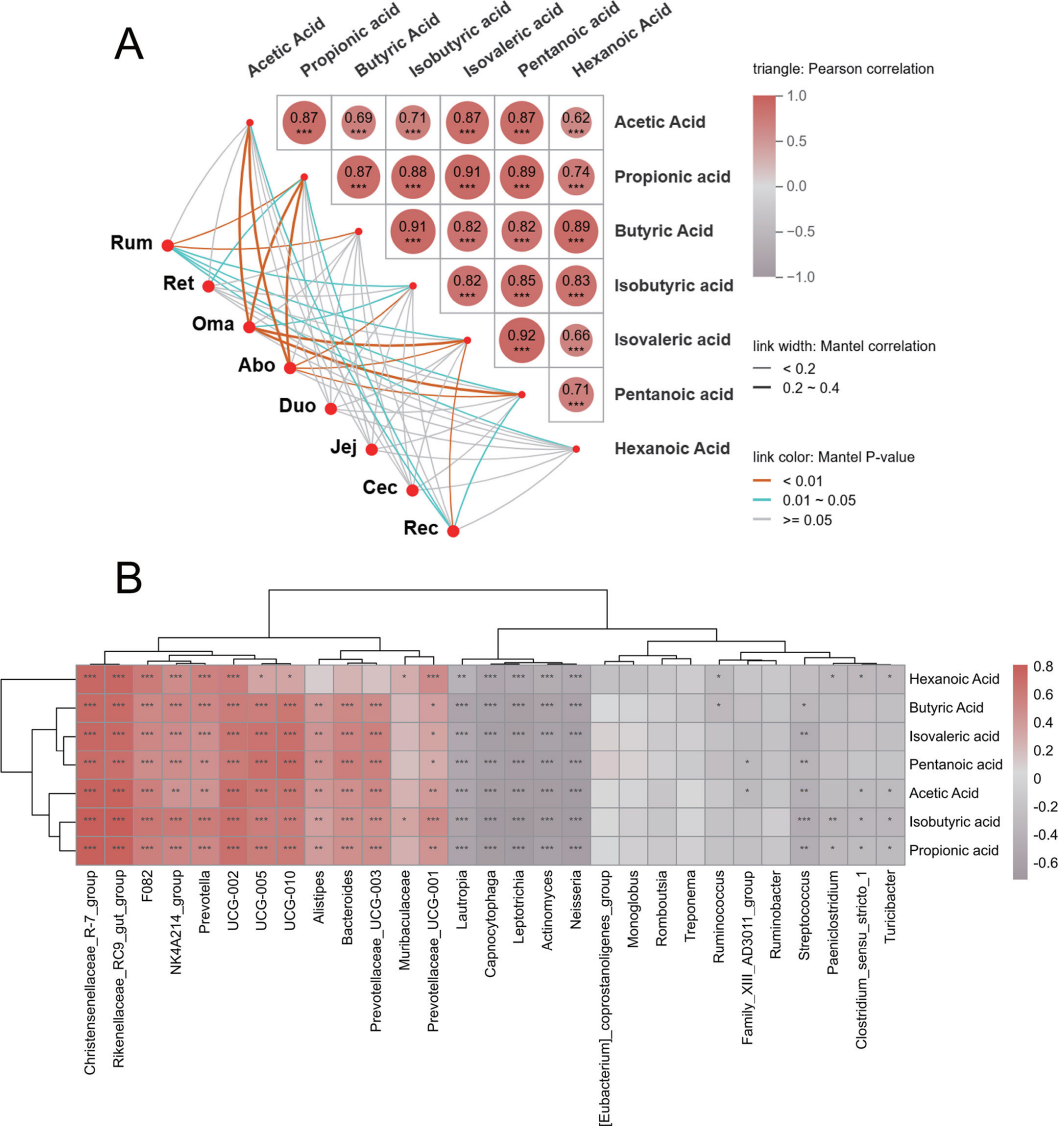
(**A**) Pearson correlation analysis between different VFAs and Mantel correlation network diagram between microbial communities in various parts of the GIT and VFAs. The upper right corner is a Pearson correlation heat map between VFAs. In the upper right corner, a Pearson correlation heat map displays pairwise correlations among VFAs. The color intensity reflects the correlation strength, with red indicating positive correlations and gray indicating negative correlations. Significance levels: *P < 0.05, **P < 0.01, and ***P < 0.001. In the lower left corner, a Mantel-based correlation network illustrates associations between microbial communities and VFAs. Red nodes represent distinct GIT segments, while blue nodes represent various VFAs. Edge colors indicate the *P*-values from the Mantel test: orange for *P* < 0.01, cyan for 0.01 ≤ *P* < 0.05, and gray for *P* ≥ 0.05. Edge thickness reflects the magnitude of the Mantel correlation coefficient: thick edges represent 0.2 ≤ *r* < 0.4, and thin edges represent *r* < 0.2. (**B**) Correlation heatmap between VFAs and key gut microbial genera. The horizontal axis represents microbial genera, and the vertical axis corresponds to VFA types. Color intensity indicates the correlation coefficient, with red denoting stronger positive correlations and gray indicating stronger negative correlations. Significance levels: *P < 0.05, **P < 0.01, and ***P < 0.001.

To explore the correlation between intestinal microbial community composition and VFA metabolism, a Mantel test was performed to evaluate the association between microbial community structure and VFA profiles across different GIT segments (Fig. 7A). Pearson’s correlation analysis revealed significant positive correlations among several VFAs. Notably, a strong correlation was observed between propionic acid and isovaleric acid (*r* = 0.91, *P* < 0.001), as well as between propionic acid and isobutyric acid (*r* = 0.91, *P* < 0.001). Additionally, isovaleric acid and pentanoic acid showed a highly significant correlation (*r* = 0.92, *P* < 0.001). These findings suggest potential metabolic synergy among these VFAs. Mantel test further revealed that the microbial communities in the Ru, Oma, Abo, and Rec were significantly positively correlated with various VFAs (*P* < 0.01). In contrast, weaker correlations were observed in the Ret, Jej, colon, and Cec. Subsequently, to explore the potential role of key intestinal bacterial genera in VFA metabolism, we further performed Spearman correlation analysis between VFAs and significantly different microbial genera and drew a correlation heat map (Fig. 7B). The results showed that most microbial genera were significantly correlated with VFAs, among which *Christensenellaceae_R-7_group*, *Rikenellaceae_RC9_gut_group*, *F082*, *NK4A214_group,* and *Prevotella* showed a significant negative correlation (*P* < 0.05). It is worth noting that, on the contrary, *Christensenellaceae_R-7_group* and *Rikenellaceae_RC9_gut_group* were most significantly correlated with VFAs. Among them, *Lautropia*, *Capnocytophaga*, *Leptotrichia*, *Actinomyces,* and *Neisseria* were extremely significantly negatively correlated with acetic acid, butyric acid, isovaleric acid, etc. (*P* < 0.001).

## DISCUSSION

This study systematically analyzed the microbial community structure, VFA distribution, and functional pathway characteristics across different regions of the GIT in Pamir yaks. The primary objective was to elucidate the spatial heterogeneity of fermentative metabolic functions in various regions of the digestive tract of high-altitude ruminants. VFAs exhibited a pronounced spatial gradient along the GIT of Pamir yaks. The highest concentrations were observed in the forestomach regions, including the Rum, Ret, and Oma. A significant decrease was detected in the Abo and small intestine, followed by a subsequent increase in the hindgut, where a “second peak” was observed. This distribution pattern was closely associated with the dominant genera and key metabolic pathways, suggesting that different regions of the intestine perform distinct roles in energy acquisition and metabolic regulation. Taken together, these findings provided new evidence for the adaptive mechanisms of energy metabolism in yaks living under extreme high-altitude environments.

Further integrating microbial community composition with functional pathway profiles, we found that *Prevotella* was a key participant in ruminal metabolism, playing a central role in carbohydrate fermentation and propionate production (34, 35). In addition, the genus *NK4A214_group* has been shown to play a key role in the degradation of cellulose and hemicellulose. It was involved in the breakdown of plant fibers, leading to the production of VFAs, including acetic acid, propionic acid, and butyric acid. This process serves as an important energy source for the host (36–38). Consistently, the enrichment of metabolic pathways, including pentose and glucuronate interconversions as well as carbohydrate digestion and absorption, was consistently observed across the samples. This finding further indicated that ruminal microbiota possess a strong capacity for the depolymerization of plant cell wall polysaccharides and the transformation of phenolic compounds. Previous studies have reported that *F082* was closely associated with propionate metabolism. Its metabolic products have been shown to modulate rumen epithelial function. This regulatory effect might help yaks optimize energy acquisition in cold, high-altitude environments (39). Interestingly, our functional prediction analysis revealed that the propanoate metabolism pathway was significantly enriched in the Ret. *F082* was identified as the only microbial biomarker in the Ret of Pamir yaks. We therefore speculated that it may contribute to efficient VFA production and absorption in this critical transitional compartment by promoting propionate synthesis and enhancing the local fermentation environment. This functional role could ultimately support host energy balance and adaptation to extreme environments. As a crucial transitional compartment linking the Ret and Abo, the ma harbors a microbial community with a distinct compositional pattern. *Prevotellaceae UCG-001* is a member of the family *Prevotellaceae* and is widely distributed in the GIT of ruminants. It exhibits strong metabolic potential for the degradation and utilization of complex carbohydrates. Previous studies have shown that the abundance of this microorganism in the ruminal mucosa of goats gradually increases with host development. This trend suggested that it not only adapts to the local mucosal environment but also participates in nutrient metabolism at this site (40). Moreover, *Prevotellaceae UCG-001* was capable of producing propionate and butyric acid through polysaccharide metabolism, thereby providing an energy source for the mucosal epithelium (41). This function might be of particular importance for energy regulation in the Oma as a transitional compartment. In addition, *Rikenellaceae_RC9_gut_group* has been identified as a key microbial taxon involved in regulating acetate metabolism and might potentially influence glucose and lipid metabolism in host muscle tissue (42). In the present study, the relative abundance of this genus was significantly higher in the forestomach (Rum and Ret) and hindgut (Cec and Rec) compared to the bo and small intestine. Furthermore, its spatial distribution pattern was consistent with the variation in acetate concentrations across these gastrointestinal segments. Based on the results of functional prediction analysis, we found that the relative abundance of various metabolic pathways in the Oma was lower than that in the Ret and Abo. This finding indicated that the metabolic activity of the Oma exhibits transitional characteristics, positioned between the Ret and Abo. It might play a pivotal role in energy regulation and in the transport of fermentation products. These results indicated that, even under extreme high-altitude conditions, the ruminal microbiota of Pamir yaks may act synergistically to promote fiber and starch degradation as well as amino acid metabolism. This microbial activity also supported the production and utilization of VFAs. Together, these processes contributed to efficient energy acquisition in high-altitude environments. Studies have shown that there is a significant difference in pH values between the Abo and the Rum. Under conditions of high-concentration feed challenge, the pH value of the Abo was significantly higher than that of the Rum (43, 44). In addition, the primary function of the Abo was to transfer smaller particles to the Oma through biphasic contractions. Larger particles were redirected to the Rum for further digestion. These physiological characteristics might result in relatively limited fermentative activity in the Abo, thereby affecting its VFA production and concentration (45). This finding explained the marked decline in VFA concentrations from the Rum to the Abo. It also provided important context for understanding the further reduction in VFAs and the characteristics of the microbial community observed in the subsequent small intestine.

In the forestomach, microbial communities primarily contribute to the host’s energy metabolism through the fermentation of fibrous forage and the production of VFAs. However, upon entering the small intestine, the digestive environment undergoes substantial changes. These included elevated gastric acid secretion, increased digestive enzyme activity, accelerated peristalsis, and reduced availability of fermentable substrates. Collectively, these alterations resulted in a significant decrease in SCFA concentrations (43). This indicated that the small intestine plays a functionally distinct role compared to the forestomach in nutrient transport and microbial regulation. In our study, we observed that the duodenum contains relatively low concentrations of SCFAs. Despite this, it maintained comparatively high microbial diversity and was enriched with multiple characteristic microbial taxa. For example, *Ruminococcus gnavus* can degrade mucin and modify bile acids. It delivered its metabolic products to the liver via the portal vein, thereby contributing to the regulation of glucose and lipid metabolism (46). At the same time, pathways such as pyrimidine metabolism, nucleotide metabolism, and galactose metabolism were significantly upregulated. These findings indicated that both protein synthesis and carbohydrate utilization remain comparatively active in the Duo. Upon entry into the Jej, the digestive environment might become more stringent. In contrast, the microbial community composition in the Jej was markedly simplified. Only four significant biomarker genera were detected in this study, and a significant reduction was observed in multiple metabolic pathways. These findings might further suggest that the jejunal microbiota experiences a metabolically constrained state under conditions of limited substrate availability and inhibitory digestive factors. As chyme progresses further along the GIT, undigested carbohydrates and protein reach the hindgut that comprises the Cec and colon. These compounds serve as novel fermentable substrates for the resident microbiota (47). Studies have shown that SCFAs produced by cecal and colonic fermentation contribute approximately 10%–15% of the metabolizable energy in ruminants (48). This finding was consistent with the “second peak” of SCFA concentrations observed in this study. Moreover, the results of correlation analysis further confirmed the spatial gradient distribution pattern of VFAs across different GIT segments.

Although this study systematically characterized the microbial community structure and VFA distribution patterns in the GIT of Pamir yaks, certain limitations remain. The sample size in this study was relatively limited, encompassing yaks from specific sex and age groups only. Furthermore, microbial functional analysis was primarily based on PICRUSt2 predictions derived from 16S rRNA gene sequencing data. These predictions were not validated using complementary metagenomic or metatranscriptomic approaches. As a result, interpretations of key metabolic pathways and functional profiles remain tentative. Future studies should prioritize expanding the sample size and integrating metagenomic and metatranscriptomic methodologies to more rigorously validate and refine the findings of this research.

### Conclusion

This study systematically analyzed the microbial communities and VFA metabolism across different regions of the GIT in Pamir yaks. The results revealed significant spatial heterogeneity. The forestomach exhibited the highest concentrations of VFAs. It was enriched in acid-producing bacteria, which underscores its role as a major energy source for the host. Although microbial diversity is relatively low in the small intestine, its metabolic functions appear to be more active. The hindgut displayed specific bacterial colonization and potential compensatory metabolic features. Functional predictions suggested a partitioning trend of metabolic pathways from the forestomach to the colon. Correlation analysis between microorganisms and VFAs indicated that microbial communities in different segments might influence the accumulation and transformation of VFAs through synergistic or antagonistic interactions. Overall, this study revealed the spatial differentiation of microbial communities and metabolic functions in the GIT of Pamir yaks. It also suggested that such regional specialization might contribute to energy acquisition and nutritional adaptation under high-altitude conditions. However, this hypothesis requires further functional validation.

## Data Availability

The data sets presented in this study can be found in online repositories. All raw sequence data were deposited in the NCBI Sequence Read Archive database under accession number PRJNA1268167.

## AFTER EPUB

*[This article was published on 25 February 2025 with errors in the Acknowledgements. The funding information in the Acknowledgements was corrected in the current version, posted on 10 March 2026.]*
